# Nigella A ameliorates inflammation and intestinal flora imbalance in DSS induced colitis mice

**DOI:** 10.1186/s13568-020-01114-3

**Published:** 2020-10-04

**Authors:** Xingjiang Hu, Nana Xu, Xi Yang, Xi Hu, Yunliang Zheng, Qiao Zhang

**Affiliations:** Zhejiang Provincial Key Laboratory for Drug Evaluation and Clinical Research, Department of Clinical Pharmacy, The First Affiliated Hospital, Zhejiang University School of Medicine, 310003 Hangzhou, People’s Republic of China

**Keywords:** Nigella A, Microbiota, Ulcerative colitis, *Nigella glandulifera*

## Abstract

Nigella A, also named Sieboldianoside A, has been extracted from many kinds of Traditional Chinese Medicine (TCM), such as *Nigella glandulifera*, *Stauntonia chinensis* DC., and the leaves of *Acanthopanax sieboldianus*. Nigella A exhibited potential analgesic, anti-inflammatory, anti-tumor, and antioxidant activities. However, whether Nigella A could treat ulcerative colitis (UC) is still unknown. As saponins always be regarded as the kinds of ingredients that could regulate immunity and intestinal flora. This research aimed to investigate the therapeutic effect of Nigella A on UC and explore its effect on intestinal flora. We noted that Nigella A and Sulfasalazine (SASP) could significantly improve the signs and symptoms, alleviate colonic pathological injury in DSS-induced mice. The changing of many specific bacterial genus such as *Lactobacillus*,* Porphyromonadaceae*,* Bacteroides* and *Escherichia* might closely related to the recovery of intestinal inflammatory response. This study initially confirmed the therapeutic effect of Nigella A and SASP on DSS-induced colitis by improving the diversity of intestinal microbial composition. Nigella A has the potential to be developed for the treatment of UC and other disorders related to the imbalance of intestinal flora.

## Key points

1. We firstly confirmed the anti-ulcerative colitis effects of Nigella A in vivo.
2. Nigella A and SASP could significantly improve the disorders of intestinal flora.
3. Nigella A showed similar activity and mechanism of action to that of SASP.

## Introduction

Inflammatory bowel disease (IBD) is an idiopathic disease that affects the ileum, rectum and colon, including ulcerative colitis (UC) and crohn’s disease (CD). UC is a kind of serious disease of digestive system with high incidence worldwide (Ng et al. 2018). Inflammation, intestinal flora, and immunity were strongly related to the occurrence and recovery of UC (Frosali et al. 2015). The interaction between the intestinal microbial community and the mucosal immune system has been identified as the key to chronic inflammation, and the changes of diversity and composition of intestinal microbiome may play an important role in the therapeutic of IBD (Cani and Delzenne 2009; Coqueiro et al. 2019). Previous results suggested that saponins are the kinds of ingredients that could regulate intestinal inflammation in IBD by the mechanisms of regulating immunity and intestinal flora (Dong et al. 2019; Guo et al. 2019).

The seeds of *Nigella glandulifera* Freyn et Sint (*N. glandulifera*), which were known as black cumin (black seeds) and were widely used as a traditional medicine for the treatment of numerous inflammatory diseases (Zheng et al. 2020). *N. glandulifera* seed powder, extracts (ethanolic, volatile oil, total flavonoids, and total saponins, etc.), and some active ingredients showed significant anti-inflammatory effects and might be effective against many kinds of inflammatory diseases (Zheng et al. 2020). For example, the total saponins from *N. glandulifera* (TSN) exhibited potential analgesic, anti-inflammatory, anti-tumor, and antioxidant activities. The content of Nigella A was 60.36 ± 1.25 g/100 g existed in TSN (Zhao et al. 2013). Our previous results revealed that saponins were the principal active components of *N. glandulifera* with the highest content (64.5%) and Nigella A (also named Sieboldianoside A) is a major oleanane triterpenoid saponin isolated from *N. glandulifera* (Zheng et al. 2020). We speculated that it could be used to treat a variety of inflammation-related diseases by regulating flora and immune.

Although the anti-inflammatory activity of *N. glandulifera* and its main constitutes have gained a great deal of attention in recent years, no research has been conducted on its anti-colitis activity. In our previous study, a large amount of Nigella A was extracted and purified from the seeds of *N. glandulifera* (Hu et al. 2014; Zheng et al. 2020)*.* Due to these healthcare functions, it is worthwhile to explore the therapeutical effect of Nigella A, a main active triterpene saponin extracted from *N. glandulifera*, on UC. Meanwhile, the mechanisms of action remain unclear and saponins always be regarded as the kinds of ingredients that could regulate immunity and intestinal flora in vivo. Based on our previous achievements, this study aimed to investigate the effects of Nigella A on dextran sodium sulfate (DSS) induced UC mice and the regulating effect of intestinal flora using modern of pharmacological techniques.

## Material and methods

### Drugs and reagents

Similar to our previous research (Zhang et al. 2020), DSS was bought from MP Biomedicals (MP Biomedicals, USA). Nigella A was isolated and purified from *N. glandulifera* in our lab with the purity of 98.4% (Hu et al. 2014), and its structure was confirmed by MS, 1H and ^13^C NMR spectra.

### The design of experiments

As we previously reported without any changes (Zhang et al. 2020), thirty male C57BL/6 SPF mice were obtained from the Experimental Animal Center of Zhejiang Province (Hangzhou, China). All mice were adaptability raised in 18 to 23 ℃ temperature, light/dark cycle environment after randomly divided into five groups with six mice each. Mice of the control group were free to drink pure water, other groups were allowed to drink freely of 4% DSS solution for 7 days (every 2 days replace new configuration DSS solution) to induce inflammatory bowel disease. The corresponding drugs were given to the stomach twice a day at the same time according to the dosing volume of 0.1 mL/10 g for 7 days. Based on the reported results and our preliminary experimental results, mice treated with Nigella A at 100 mg/kg for 7 days didn’t showed any symptom of toxicity or mortality. So, the optimal dosages of Nigella A (50, 100 mg/kg) and Sulfasalazine (SASP, 200 mg/kg) were chosen in animal experiments. The control and DSS groups were given of normal saline, and all mice were sacrificed for cervical dislocation on the 8th day for anatomical sampling.

### The evaluation of anti-UC effects

Disease activity index (DAI) score is a common index to evaluate the colon injury model of experimental animals. It can be used to evaluate the body weight loss, diarrhea, blood in the stool and other uncomfortable symptoms in mice after the treatment of DSS. Therefore, as we reported before, the body weight, fecal characteristics and blood in the stool of mice were evaluated in a blind manner every day according to the DAI scoring system (Zhang et al. 2020). On the 8th day of the experiment, after the colon was dissected, pathological changes such as edema, adhesion, ulcer and necrosis were observed. The length of the colon was measured with a ruler and photographed. According to the above two indicators, the efficacy of Nigella A on UC was preliminarily determined.

### The evaluation of pathological features of colon tissues

The colon tissues were isolated, measured and photographed as quickly as possible, and a part of the colon tissue was fixed with 4% paraformaldehyde for 2 days. Then, the colon fragments were embedded, sectioned, stained and observed under the microscope. The histological score was evaluated according to the scoring criteria as we previously reported (Zhang et al. 2020). The sum of each score was used to evaluate colon histopathological improvement.

### Gut microbiota analysis

#### DNA extractions

DNA from different groups was extracted using the E.Z.N.A. ^®^Stool DNA Kit (D4015, Omega, Inc., USA). Nuclear-free water was used as blank. Total DNA was eluted using 50 µL of Elution buffer for the measurement of PCR by LC-Bio Technology (Co., Ltd, Hang Zhou, Zhejiang Province, China).

#### PCR amplification and 16S rDNA sequencing

The V4 region of small-subunit (16S) rRNA gene was amplified with primers 515F (5′-GTGYCAGCMGCCGCGGTAA-3′) and 806R (5′-GGACTACHVGGGTWTCTAAT-3′). PCR amplification was performed in a total volume of 25 µL reaction mixture containing 25 ng of template DNA, 12.5 µL PCR Premix, 2.5 µL of each primer. The PCR conditions were designed as follows: initial denaturation at 98 ℃ for 30 s; 35cycles of denaturation at 98 ℃ for 10 s, annealing at 54 ℃/52 ℃ for 30 s, and extension at 72 ℃ for 45 s; and then final extension at 72 ℃ for 10 min. The PCR products were confirmed and purified by AMPure XT beads (Beckman Coulter Genomics, Danvers, MA, USA) and quantified by Qubit (Invitrogen, USA). The amplicon pools were prepared for sequencing and the size and quantity of the amplicon library were assessed on Agilent 2100 Bioanalyzer (Agilent, USA) and with the Library Quantification Kit for Illumina (Kapa Biosciences, Woburn, MA, USA), respectively. The libraries were sequenced either on PE150 HiSeq.

### Data analysis

Samples were sequenced on the platform of Illumina HiSeq according to the manufacturer's instructions (LC-Bio). Paired-end reads was assigned, truncated and merged using FLASH. Chimeric sequences were filtered by using Vsearch software (v2.3.4). Sequences with ≥ 97% similarity were assigned to the same operational taxonomic units (OTUs) by Vsearch (v2.3.4). Representative sequences were chosen for each OTU, and taxonomic data were then assigned to each representative sequence using the RDP (Ribosomal Database Project) classifier. The differences of the dominant species in different groups, multiple sequence alignment were conducted using the mafft software (V 7.310) to investigate the phylogenetic relationship of different OTUs. All of these indices in our samples were calculated with QIIME (Version 1.8.0). The Illumina sequencing raw data have been successfully registered with the BioSample database (Submission ID: SUB7839343; BioProject ID: PRJNA649021).

### Statistical analysis

For intestinal flora analysis, FLASH 1.2.8 software was used to splicing sequences, Vsearch 2.3.4 software was used to filter chimeras and OTU clustering, QIIME 1.8.0 software was used to analyze the diversity of microflora, and R 3.4.4 (R Core team) language mapping software was used. Other experimental data were revealed as mean ± SD of three experiments. The statistical significance was assessed by one-way ANOVA followed by post hoc Tukey’s test or Student’s *t*-test when appropriate by using GraphPad 7.0 software.

## Results

### Nigella A relieved the symptoms of DSS-induced UC mice

**Fig. 1:**
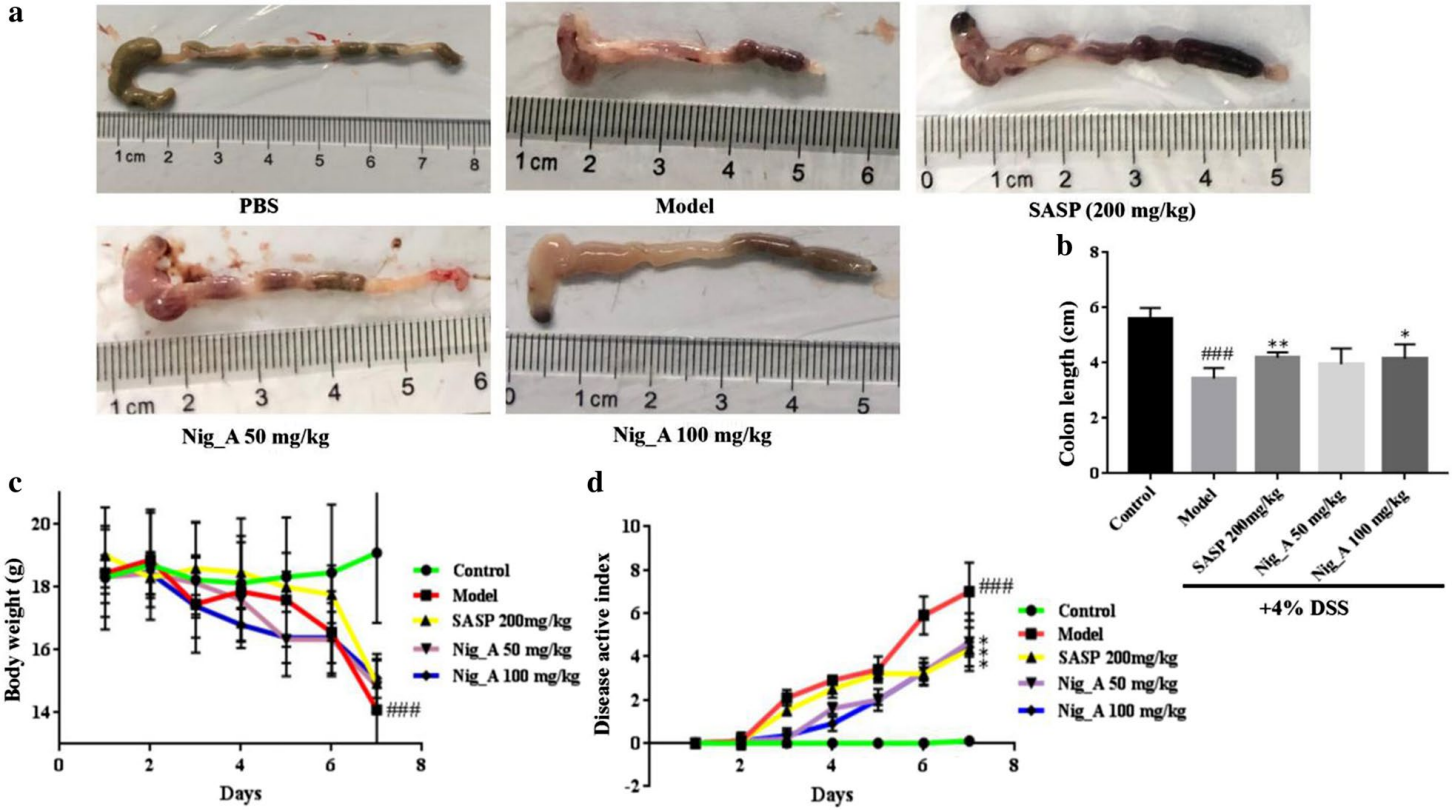
The efficiency of Nigella A in colitis tissues. **a** Nigella A effectively improved the shorten of the colon tissue. **b** Quantitative results of colon length in different groups. **c** Changes in body weight in mice of different groups over the whole course of this experiment. **d** DAI change in different experimental groups. Each point represented the mean ± SD (n = 6). ^###^*p* < 0.001 vs. control, *p < 0.05 and **p < 0.01

During continuous administration, mice of the control group grew well and slowly gained weight, while mice of DSS group and each drug intervention group lost weight, among which the DSS group had the highest degree of decline at the 8th day. During the administration period (2–8 days), mice that were given Nigella A showed a rapid weight loss from day 2 to day 6 when compared with the DSS group. The rapid weight loss of DSS group occurred on the day 5 to day 7. The weight loss of mice and the shorten of colon length were both improved. Colon length is one of main parameters to evaluate the severity of colitis. The reduction of colon length was approximately 23.8%, 13.1%, and 15.6% in colon length in DSS group, Nigella A groups and SASP group when compared with the normal group (Fig. 1a, b). Furthermore, the DAI scores were significantly increased in mice treated with Nigella A and SASP (Fig. 1c).

### Nigella A alleviated the colonic injury of DSS-induced colitis mice

**Fig. 2:**
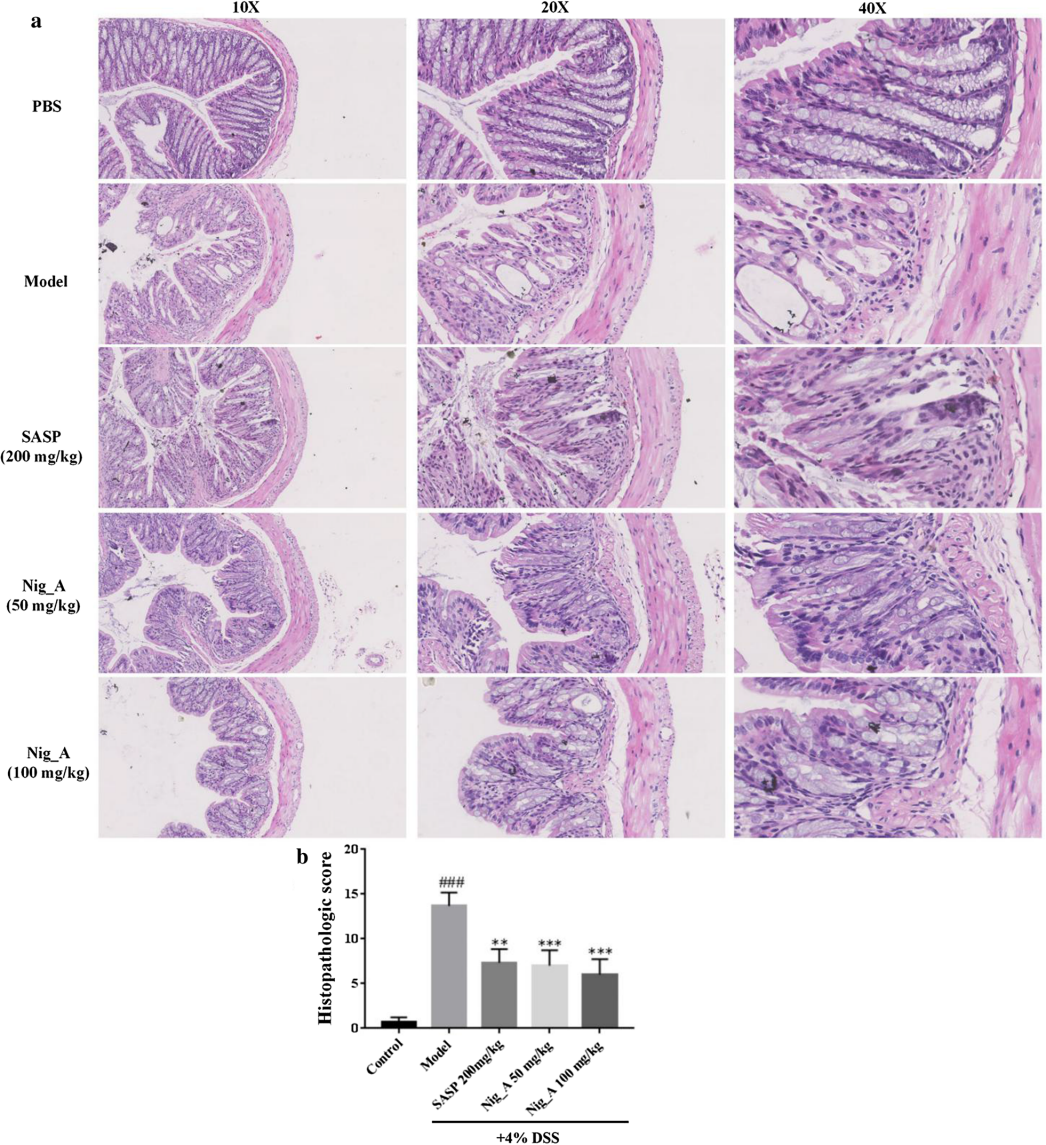
H&E-stained of representative cross-sectional colon sections in DSS-treated mice after the treatment of Nigella A and SASP for 7 days. **a** Representative pictures of colonic segments. **b** Morphology score of colon sections

The colon structure of mice in the normal group was intact, and the distribution between mucosa, submucosa, muscularis and outer membrane was clear and complete (Fig. 2a). In the model group, colonic injury, such as the erosion of epithelial monolayer, crypt loss and infiltration of immune cells, etc. were observed in submucosa and muscle layer of the colon, and the histopathologic score was significantly increased than that of the control group (Fig. 2b). The inflammatory infiltration in SASP (200 mg/kg) and Nigella A (50 and 100 mg/kg) group was slightly improved. They significantly ameliorated the damages of colonic inflammation and improve the integrity of mucosal layer. Compared with the DSS group, the histopathological score of SASP and Nigella A groups were significantly decreased. The efficacy of Nigella A is better than that of SASP, especially the high dosage of Nigella A.

### Microbial diversity analysis

#### ɑ-Diversity analysis

**Fig. 3:**
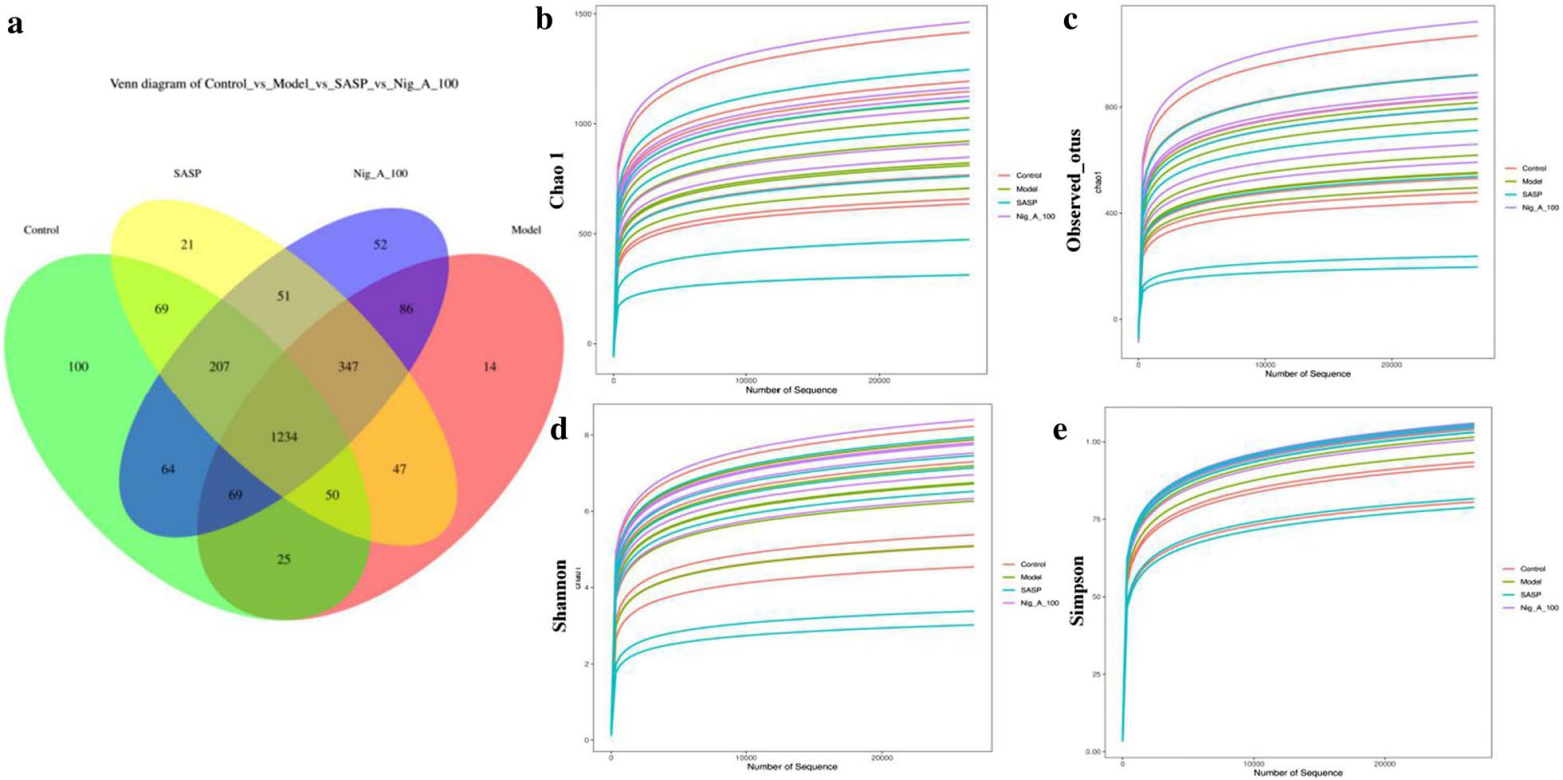
Analysis ɑ-diversity of the differential microbial community among the control group, model group, SASP and Nig_A_100-treated group. **a** Venn diagram; **b** Chao1; **c** observed species index; **d** Shannon; **e** Simpson

As shown in the Venn diagram of Fig. 3a, 1234 OTUs appeared in all groups, while 25 OTUs overlapped in the control and model groups, suggesting that DSS could significantly decrease the diversity of bacteria. 69 OTUs overlapped in the control and SASP groups, suggesting that the positive drug SASP could evaluate the diversity of bacteria to some extent. 64 OTUs overlapped in the control group and Nigella A (100 mg/kg) group (Nig_A_100), suggesting that Nig_A_100 showed similar improvement of bacteria diversity to SASP. The OTUs of control group, model group, SASP group and Nig_A_100 were 100, 14, 21 and 52, respectively. These results suggested that the diversity of intestinal flora was significantly decreased in mice induced by DSS, and the diversity of intestinal flora in SASP group and Nig_A_100 group were improved to some extent.

The rarefaction curves tended to reach the saturated plateau, which indicated that the sequencing coverage is sufficient for further data analysis. As is shown in Fig. 3b and Table 1, DSS administration repressed the microbial richness (Chao1) when compared with healthy mice. The results from the observed species index showed there was changes in alpha diversity between the control group and the model group (Fig. 3c). Meanwhile, it was largely reversed in alpha diversity after the treatment of SASP and Nig_A_100, even though the Shannon and Simpson indices of these four groups were similar (Fig. 3d, e). These results showed that SASP and Nig_A_100 could significantly improve the microbial diversity in DSS-treated mice.

**Table 1:** The results of alpha_diversity analysis

	Control	Model	SASP	Nig_A_100
Chao1	1253.20 ± 296.72	1173.89 ± 196.04	1126.78 ± 486.91	1394.07 ± 167.87
Observed_otus	1010.67 ± 285.73	958.50 ± 181.45	903.17 ± 440.10	1182.00 ± 180.18
Shannon	6.02 ± 1.49	6.24 ± 0.88	5.55 ± 2.03	7.02 ± 0.68
Simpson	0.90 ± 0.09	0.96 ± 0.03	0.90 ± 0.12	0.97 ± 0.02

**Fig. 4:**
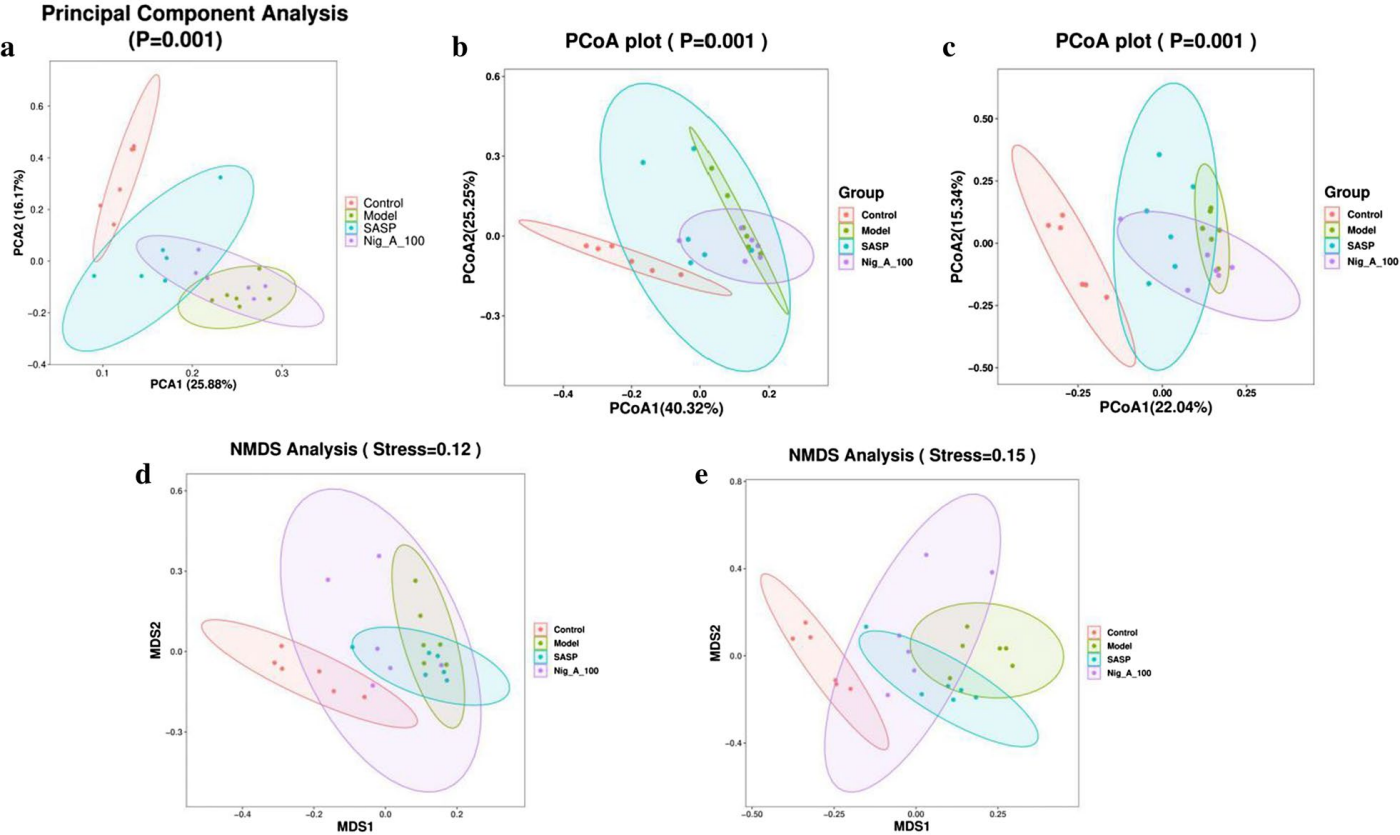
Analysis of bacterial community diversity of intestinal contents of different groups. **a** Unifrac distance of weighted PCoA analysis. **b** Unifrac distance of unweighted PCoA analysis; **c** Unifrac distance of weighted NMDS analysis. **d** Unifrac distance of unweighted NMDS analysis

#### β-Diversity analysis

The results of principal component analysis (Fig. 4a), principal coordinate analysis (PCoA) of weighted (Fig. 4b) and unweighted Unifrac distance (Fig. 4c) suggested that no overlap between the control group and model group, indicating that the number of differential OTUs was high. However, SASP and Nig_A_100 groups were more inclined to the control group, suggesting that SASP group and Nig_A_100 groups had the function of regulating the tendency of flora to normal. The results of non-metric multi-dimensional scaling (NMDS) suggested that model group and control group showed significant isolated clusters, indicating a variation tendency of the altered main microbial composition structures (Fig. 4d, e). However, the community structure of SASP and Nig_A_100 groups were significantly inclined to the control group.

#### Cluster analysis

**Fig. 5:**
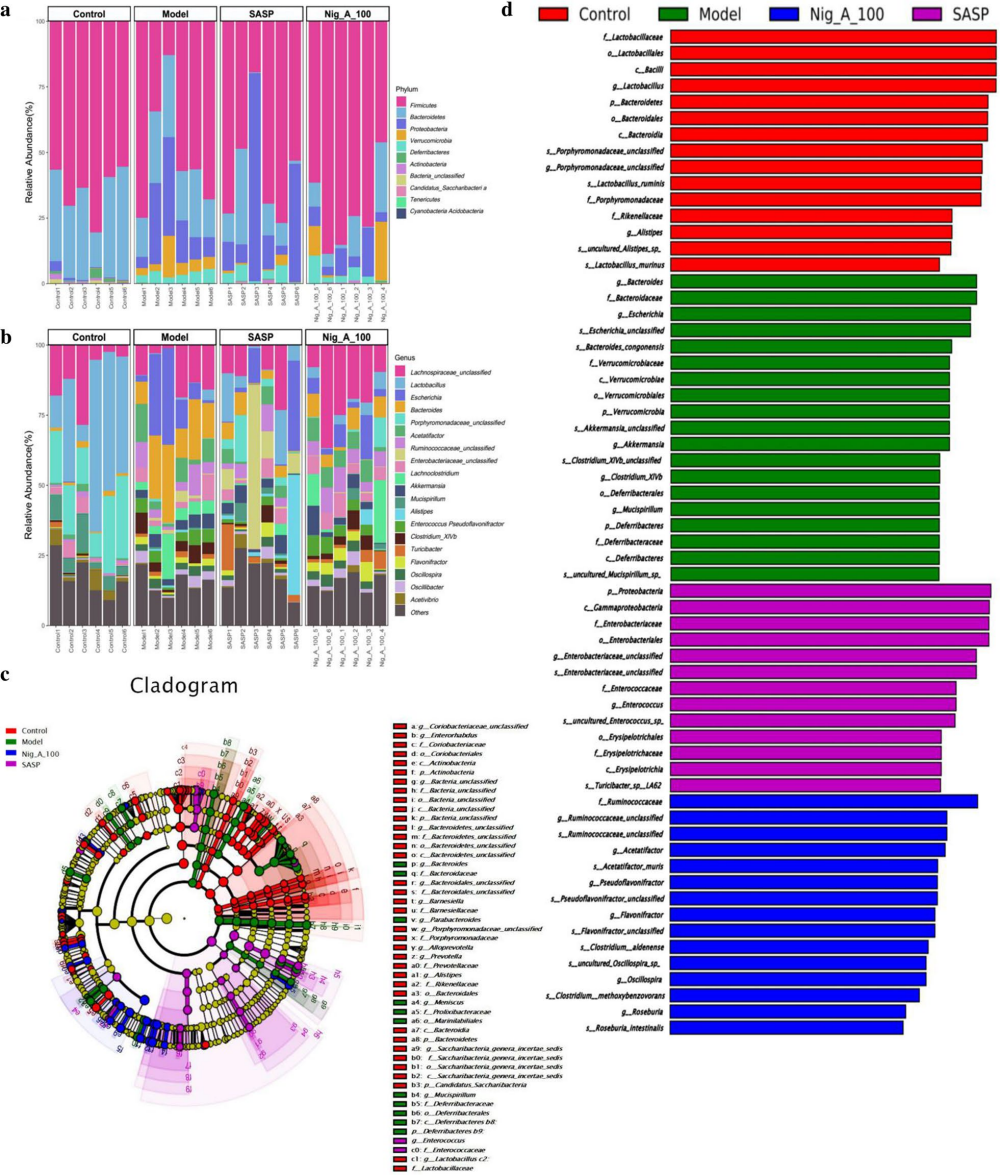
Structural comparison of fecal flora among the control, model, SASP and Nig_A_100 groups. Bacterial taxonomic profiling at the phylum lever (**a**) and genus level (**b**). LEfSe analysis of fecal flora among the control, model, SASP and Nig_A_100 groups. **c** LEfSe analysis showed the distribution histogram of different groups in gut microbiota (LDA sore > 4). **d** Cladogram. The size of each node represents the relative abundance of the species. (p, phylum; c, class; o, order; f, family; g, genus; s, species)

Metastatistical analysis showed that, at the phylum level, there were three phyla, including *Firmicutes*, *Bacteria_unclassified* and *Proteobacteria*, that displayed significant differences in the relative abundance between the model group and the control group (Table 2). These three phyla in the model group exhibited significant differences of the relative abundance with SASP and Nig_A_100 group (Fig. 5a). At the genus level, the changes of model group were observed in many bacterial genus (Fig. 5b). The taxonomic profiling showed a significant lower proportion of *Lactobacillus*, *Porphyromonadaceae_unclassified*, *Alistipes*, *Acetivibrio*, *Barnesiella*,* Bacteria_unclassified* and an a significant increase of *Parabacteroides*,* Desulfovibrio*, *Romboutsia*, *Bacteroides*, *Escherichia*, *Clostridium_XlVb*, *Meniscus* in the model when compared with the control group (Table 3). However, Nig_A_100 and SASP significantly reversed the levels of these changes. In addition, our results showed that there were significant changes in up to 61 genera after DSS treatment, and most of the changes could be reversed by SASP and Nig_A_100 (Additional file 1: Table S1 and Additional file 2: Figure S1). These results showed that at least at the level of genus, Nig_A_100 and SASP could significantly regulate the changes of a variety of bacterins and then affect the intestinal flora in the model of ulcerative colitis mice.

**Table 2:** Cluster analysis of phylum level

Phylum	Control	Model	SASP	Nig_A_100
*p_Candidatus_Saccharibacteria*	0.62	0.01	0.02	0.04
*p_Proteobacteria*	1.04	17.46*	26.52	7.87^#^
*p_Deferribacteres*	0.07	3.82	3.52	3.75
*p_Verrucomicrobia*	0.07	5.87	1.01	5.83
*p_Bacteroidetes*	32.02	22.07	11.56	9.69
*p_Bacteria_unclassified*	0.73	0.04*	0.27^#^	0.28^#^
*p_Actinobacteria*	1.15	0.08	0.12	0.14
*p_Firmicutes*	64.24	50.56*	56.75	72.25^#^

*p < 0.05 vs. control, ^#^*p* < 0.05 vs. model

**Table 3:** Cluster analysis of main bacterial genus levels

Genus	Control	Model	SASP	Nig_A_100
*g_Lactobacillus*	34.35	0.88*	7.84^#^	3.22^#^
*g_Porphyromonadaceae_unclassified*	19.03	0.25*	4.33^#^	1.97^#^
*g_Alistipes*	6.37	0.34*	1.70^#^	1.07^#^
*g_Acetivibrio*	3.33	0.16*	1.39^#^	0.63^#^
*g_Barnesiella*	2.88	0.03*	0.91^#^	0.78^#^
*g_Bacteroides*	1.31	17.19*	3.66^#^	4.93^#^
*g_Bacteria_unclassified*	0.73	0.04*	0.27^#^	0.28^#^
*g_Clostridium_XlVb*	0.35	4.30*	1.46^#^	3.10^#^
*g_Escherichia*	0.10	13.74*	7.84	5.98^#^
*g_Parabacteroides*	0.04	0.86*	0.33^#^	0.22^#^
*g_Desulfovibrio*	0.03	0.47*	0.20^#^	0.08^#^
*g_Romboutsia*	0.02	1.92*	0.39^#^	0.70^#^
*g_Meniscus*	0.02	3.18*	0.40^#^	0.10^#^

*p < 0.05 vs. control, ^#^*p* < 0.05 vs. model

### Overall structure modulation of gut microbiota after Nig_A_100 treatment

Consistent with other results, the cladogram, generated from the linear discriminant analysis effect size (LEfSe) analysis, showed distinct gut microbiota compositions among mice from all groups (Fig. 5c, d). Great changes have happened of dominant bacterial taxa after the intervention of DSS. The comparison of dominant bacterial taxa at genus level demonstrated that DSS increased the relative abundance of *p__Bacteroidetes/c__Bacteroidia/o__Bacteroidales/f__Bacteroidaceae/g__Bacteroides* and *p__Proteobacteria/c__Gammaproteobacteria/o__Enterobacteriales/f__Enterobacteriaceae/g__Escherichia.* Meanwhile, DSS decreased the relative abundance of *p__Firmicutes/c__Bacilli/o__Lactobacillales/f__Lactobacillaceae/g__Lactobacillus* and *p__Bacteroidetes/c__Bacteroidia/o__Bacteroidales/f__Porphyromonadaceae/g__Porphyromonadaceae_unclassified*. However, the abundances of these main bacterial genus were significantly reversed by Nig_A_100 and SASP (Additional file 1: Table S1).

## Discussion

*Nigella glandulifera* has been widely used in Islamic countries as a kind of traditional medicinal herb and a food additive since ancient times (Zheng et al. 2020). The whole herb of *N. glandulifera* has been used for a variety of inflammatory disease, such as colds, coughs, and insomnia (Chinese Pharmacopoeia, 2015). *N. glandulifera* seeds also traditionally were used as a spice or remedy to treat various inflammatory diseases (Zheng et al. 2020). For example, *N. glandulifera* and TSN were confirmed to exhibit significant antioxidant and anti-inflammatory properties (Zhao et al. 2013; Zheng et al. 2020). Triterpene saponins are the dominant group of anti-inflammatory compounds existed in this ethnomedicinal plant (Hu et al. 2014; Zheng et al. 2020) and Nigella A was a major ingredient of triterpene saponins extracted from *N. glandulifera*. Nigella A was 60.36 ± 1.25 g/100 g total saponins of *N. glandulifera* (Zhao et al. 2013). All of these results supplied sufficient theoretical evidence for the anti-UC effect of triterpene saponins and specifically Nigella A in the therapeutic field of UC.

It has been reported that saponins always exhibited protective effects against a variety of diseases fermented with probiotic (Kim 2018; Ku 2016). Our previous review revealed that many kinds of extractions of *N. glandulifera* seeds and some active ingredients always exhibited significant anti-inflammatory potentials and might be effective on various inflammatory diseases (Zheng et al. 2020). For example, TSN could be considered to be a potential analgesic, anti-inflammatory, anti-tumor, and antioxidant agent (Zhao et al. 2013). Our present results suggested that Nigella A, a major component of triterpene saponins extracted from *N. glandulifera*, could significantly attenuate colonic shortening, induced a reduction in body weight, and led to the DAI of mice. These results basically confirmed the efficacy of Nigella A on experimental UC mice.

*Bacteroids* and *Escherichia* are highly related to gut inflammation, which are reported in several IBD animal models and patients of various literature (Yeom et al. 2016; Rattigan et al. 2020). *Lactobacillus*, as a kind of probiotic, exhibited beneficial effects on IBD by stimulating immune cells (Liu et al. 2018). *Porphyromonadaceae* always exhibited a evident depleted trend in UC patients (Zhang et al. 2019). Our results demonstrated that DSS could significantly decrease the proportion of *Lactobacillus* and *Porphyromonadaceae* and increase the proportion of *Bacteroides* and *Escherichia*. However, the maximum variation of these four bacterial genus could significantly reversed by SASP and Nigella A. The maximum variation of *Lactobacillus, Porphyromonadaceae, Bacteroides* and *Escherichia* levels suggested that these four kinds of bacterial genus may be the main bacterial genus affected by SASP and Nigella A.

In addition, we also detected a significantly up-regulated or down-regulated of a large number of other bacterial genus after the intervention of DSS. We detected significant changes of 62 genera after the intervention of DSS, and most of the changes could be reversed by SASP and Nig_A_100. As for the regulation of intestinal flora and the therapeutic effect of UC, although current experimental results can’t provide accurate correlation results, the regulation of intestinal flora is at least one of the mechanisms of SASP and Nigella A. Taken together, SASP and Nigella A could alleviate microbiota dysbiosis in DSS-induced colitis model with the involvement of intestinal flora regulation. In addition, there are many similar saponins exist in *N. glandulifera* that show similar pharmacological activities. Nigella A was only one of these representative components, but it exhibits a similar anti-UC effect to SASP. Due to the fact that Nigella A was also found in many other plants and Traditional Chinese Medicine (TCM), other saponins and TCM extractions containing Nigella A may also exhibit anti-UC effects.

It's worth mentioning that SASP has been used in clinic for many years for the therapy of UC, the regulation of intestinal flora is also one of its mechanisms of action (Neumann et al. 1987). And once again, our results proved that SASP has the functions of regulating intestinal flora in DSS induced mice. It is interesting to note that Nigella A (100 mg/kg) exhibited very similar regulatory effects of intestinal flora to SASP (200 mg/kg). Considering the similar anti-UC activities of SASP and Nigella A, as well as the multiple mechanisms of action due to SASP. We speculated that Nigella A may also have other mechanisms of action for the treatment of UC, regulating gut flora is at least one of the mechanisms by which Nigella A works. Nigella A has been widely reported to have significant anti-inflammatory and immune-regulating functions (Zheng et al. 2020), which may also play a role in the mechanisms of its anti-UC effect. All of these speculations need to be confirmed by further experiments.

In summary, the oral administration of Nigella A and SASP exhibited therapeutic effects on DSS-induced UC in mice. The therapeutic effects manifested as the improvement of the general symptoms and the remission of inflammatory injure. The underlying mechanism of action of these effects involved the preventing of intestinal flora imbalance and restoring the relative abundances of vital bacteria. These results revealed that Nigella A had a protective effect on DSS-induced UC with the involvement of regulating intestinal flora. Nigella A was considered to be a novel attractive intestinal microecological improver with great development prospects.

## Supplementary information

**Additional file 1.** The original results of 16S rDNA sequencing and statistical analysis of flora differences.

**Additional file 2.** The bubble_plot of the control, model, SASP and Nig_A_100 groups about the bacterial abundance variation at the phylum and genus levels in fecal samples.

## Data Availability

All data generated or analysed during this study are included in this published article and its Additional files 1, 2.

## Abbreviations

CD: Crohn's disease
DAI: Disease activity index
DMSO: Dimethyl sulphoxide
DSS: Dextran sulfate sodium
IBD: Inflammatory bowel disease
NMDS: Non-metric multi-dimensional scaling
OTUs: Operational taxonomic units
UC: Ulcerative colitis
PCoA: Principal coordinate analysis
SASP: Sulfasalazine
TCM: Traditional Chinese medicine
