# Supplementary material for: Nigella A ameliorates inflammation and intestinal flora imbalance in DSS induced colitis mice
Source: AMB Express. 2020 Oct 4;10:179. doi: 10.1186/s13568-020-01114-3 (PMC7533278; doi:10.1186/s13568-020-01114-3)
Supplement: Supplementary file 2 — Additional file 2. The bubble_plot of the control, model, SASP and Nig_A_100 groups about the bacterial abundance variation at the phylum and genus levels in fecal samples. [file 13568_2020_1114_MOESM2_ESM.pdf]

AMB Express

Nigella A ameliorates experimental colitis via interacting with gut microbiota on dextran sulphate sodium (DSS)-induced C57BL/6 mice

Xingjiang Hu, Nana Xu, Xi Yang, Xi Hu, Yunliang Zheng, Qiao Zhang

Department of Clinical Pharmacy, the First Affiliated Hospital, Zhejiang University School of Medicine, Hangzhou 310003, People's Republic of China

Corresponding author. Tel./fax: +0571 87236596.
E-mail: 1316056@zju.edu.cn (Qiao Zhang)

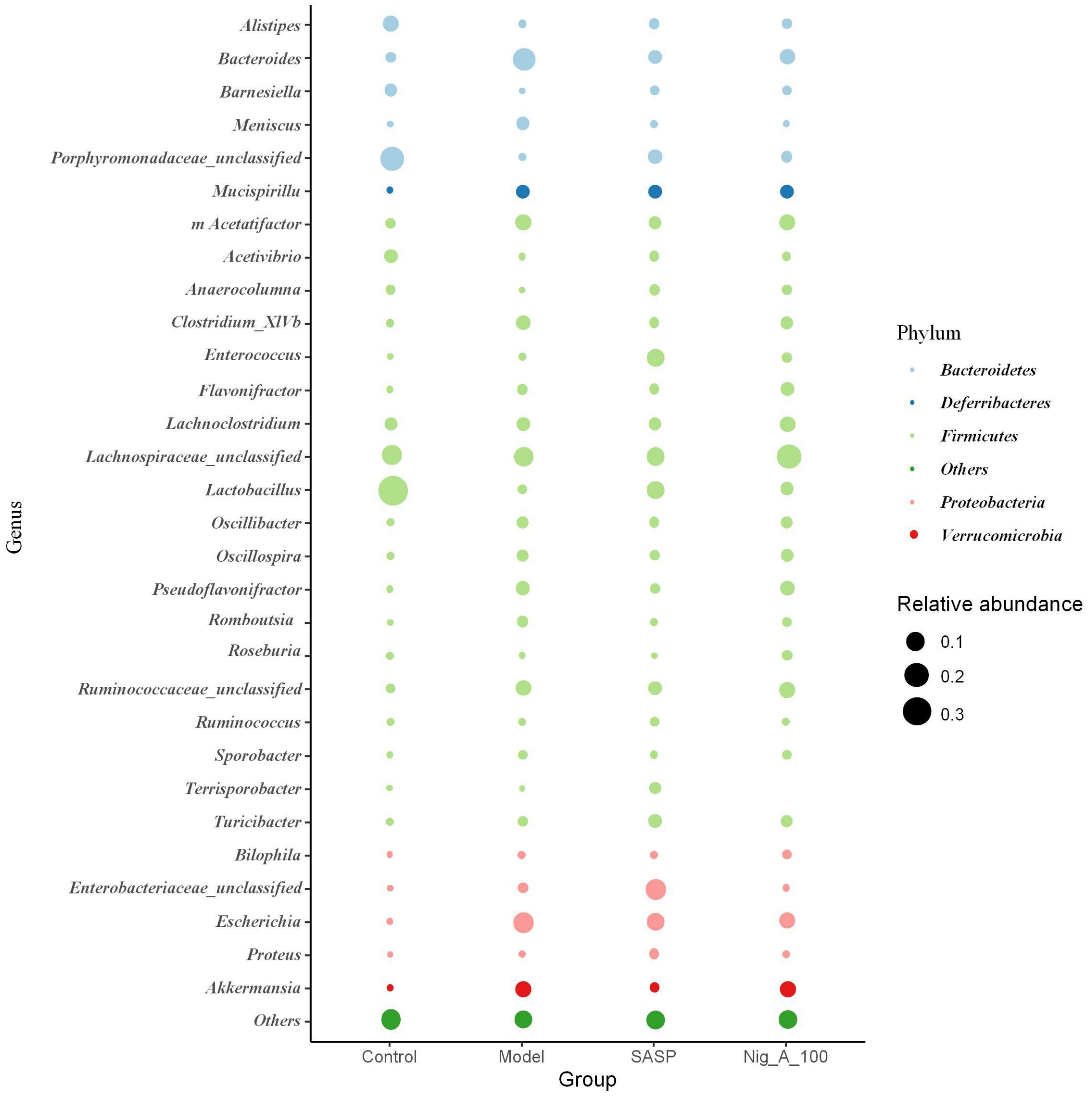

Fig. S1 The bubble_plot of the control, model, SASP and Nig_A_100 groups about the bacterial abundance variation at the phylum and genus levels in fecal samples.
